# The higher prevalence of extended spectrum beta-lactamases among *Escherichia coli* ST131 in Southeast Asia is driven by expansion of a single, locally prevalent subclone

**DOI:** 10.1038/s41598-019-49467-5

**Published:** 2019-09-13

**Authors:** Swaine L. Chen, Ying Ding, Anucha Apisarnthanarak, Shirin Kalimuddin, Sophia Archuleta, Sharifah Faridah Syed Omar, Partha Pratim De, Tse Hsien Koh, Kean Lee Chew, Nadia Atiya, Nuntra Suwantarat, Rukumani Devi Velayuthan, Joshua Guo Xian Wong, David C. Lye

**Affiliations:** 1 Genome Institute of Singapore, Agency for Science, Technology, and Research, 60 Biopolis Street, Genome #02-01, Singapore, 138672 Singapore; 2 Department of Medicine, Division of Infectious Diseases, Yong Loo Lin School of Medicine, National University of Singapore, 1E Kent Ridge Road, NUHS Tower Block, Level 10, Singapore, 119228 Singapore; 3 National Centre for Infectious Diseases, 16 Jalan Tan Tock Seng, Singapore, 308442 Singapore; 4 Division of Infectious Diseases, Faculty of Medicine, Thammasat University Hospital, 95 Phahonyothin Rd, Khlong Nueng, Khlong Luang District, Pathum Thani, 12120 Thailand; 5 Department of Microbiology, Division of Pathology, Singapore General Hospital, Academia, Diagnostics Tower, Level 7, 20 College Road, Singapore, 169856 Singapore; 6 University Medicine Cluster, Division of Infectious Diseases, National University Hospital, , 5 Lower Kent Ridge Road, Singapore, 119074 Singapore; 7 Department of Medical Microbiology, Faculty of Medicine, University of Malaya, 50603 Kuala Lumpur, Malaysia; 8 Communicable Diseases Centre, Institute of Infectious Disease and Epidemiology, Tan Tock Seng Hospital, Singapore, 308433 Singapore; 9 Duke-NUS Medical School, 8 College Road, Singapore, 169857 Singapore; 10 Chulabhorn International College of Medicine, Thammasat University, Pathum Thani, 12120 Thailand; 11 Lee Kong Chian School of Medicine, Nanyang Technological University, Singapore, 639798 Singapore; 12 Department of Infectious Diseases, Singapore General Hospital, Academia Level 3, 20 College Road, Singapore, 169856 Singapore; 13 Department of Laboratory Medicine, National University Hospital, 5 Lower Kent Ridge Road, Singapore, 119074 Singapore

**Keywords:** Bacterial genes, Bacterial infection

## Abstract

The ST131 multilocus sequence type (MLST) of *Escherichia coli* is a globally successful pathogen whose dissemination is increasing rates of antibiotic resistance. Numerous global surveys have demonstrated the pervasiveness of this clone; in some regions ST131 accounts for up to 30% of all *E*. *coli* isolates. However, many regions are underrepresented in these published surveys, including Africa, South America, and Asia. We collected consecutive bloodstream *E*. *coli* isolates from three countries in Southeast Asia; ST131 was the most common MLST type. As in other studies, the C2/H30Rx clade accounted for the majority of ST131 strains. Clinical risk factors were similar to other reported studies. However, we found that nearly all of the C2 strains in this study were closely related, forming what we denote the SEA-C2 clone. The SEA-C2 clone is enriched for strains from Asia, particularly Southeast Asia and Singapore. The SEA-C2 clone accounts for all of the excess resistance and virulence of ST131 relative to non-ST131 *E*. *coli*. The SEA-C2 strains appear to be locally circulating and dominant in Southeast Asia, despite the intuition that high international connectivity and travel would enable frequent opportunities for other strains to establish themselves.

## Introduction

*Escherichia coli* is a common member of the colonic flora of humans and other animals, where it often appears be a commensal^1^. However, *E*. *coli* also causes a variety of diseases, which are generally classified based on the disease syndromes they cause. Some of these *E*. *coli* classifications include EHEC (enterohemmorhagic *E*. *coli*), ETEC (enterotoxigenic *E*. *coli*), and UPEC (uropathogenic *E*. *coli*)^1,2^. Many authors distinguish between *E*. *coli* that are associated with intestinal and extra-intestinal (outside the gastrointestinal tract) diseases; those causing extra-intestinal disease are referred to as a group as ExPEC (extraintestinal pathogenic *E*. *coli*). ExPECs commonly cause urinary tract infections, bloodstream infections, meningitis, and soft tissue infections^2^. These ExPEC infections are typically more medically serious than the intestinal syndromes caused by *E*. *coli*, and their treatment relies on effective antibiotic therapy. Unfortunately, as in other bacteria, antibiotic resistance rates in ExPEC strains have been rising in recent years^3^. In fact, ExPEC strains are generally more antibiotic resistant than other types of *E*. *coli*, and the expansion of specific ExPEC clones or multilocus sequence types (MLSTs) have been contributing to the rising rates of *E. coli* antibiotic resistance, such as ST38, ST405, and ST648^4^.

Another of these ExPEC sequence types, ST131 *E*. *coli*, has been more extensively studied over the last 10 years and found to be rapidly expanding across the globe. ST131 was first described in 2008^5–7^ and has now been found on every continent examined; it accounts for up to 30% of all ExPEC isolates in some regions^8^. Of particular concern, ST131 strains are frequently resistant to multiple commonly prescribed antibiotics, most prominently fluoroquinolones and beta-lactamases^8,9^. More specifically, ST131 strains typically carry resistance-conferring mutations in the chromosomal *gyrA* and *parC* genes (encoding DNA gyrase and DNA topoisomerase IV, respectively, the target of fluoroquinolones)^8–10^ as well as a gene encoding a CTX-M-class extended spectrum beta-lactamase (ESBL) (particularly CTX-M-15), either on a plasmid or integrated into the chromosome^11^. Coupled with the recent spread of ST131, this means that ST131 itself has been responsible for much of the observed rise in antibiotic resistance, particularly ESBL-mediated resistance, in ExPEC globally^6,12^. In addition to driving resistance, evidence also indicates that ST131 strains may be more virulent, driving higher rates of bacteremia^13^.

Numerous studies of ST131 have been performed, many of them using whole genome sequencing^11,12,14–21^. ST131 is often referred to as a clone, or clonal group, as the strains are closely related and appear to have had a single origin^5,9,12,20^. ST131 strains have been further subclassified into 3 large clades using two closely correlated naming schemes: A/H41, B/H22, and C/H30-R^8,12^. There are further subdivisions of these clades; of most importance is the subdivision of clade C/H30-R into two subclades, one of which has a high prevalence of the CTX-M-15 ESBL gene (referred to as C2/H30-Rx)^8,12,15^. Despite the general resistance of C2/H30-Rx strains to more antibiotics, however, all of Clade C/H30-R seems to be participating in the recent global expansion of ST131^15^.

A Bayesian analysis dated the divergence of clades B and C to 1938–1958 and predicted that they arose in North America^15^. This same study dated the divergence between clades C1 and C2 to 1980 and noted that, with the exception of a cluster of GI-*selC* containing strains from the UK, there was no significant geographical clustering. The available data at that time, however, was limited in strains from South America, Africa, and Asia.

In particular, there remains limited data concerning the prevalence and molecular characteristics of *E*. *coli* ST131 in Southeast Asian countries, particularly for bacteremia, as previous studies were conducted prior to the identification of ST131^22–24^ or did not perform the molecular characterization required to identify ST131^25–28^. From a clinical point of view, due to its association with antibiotic resistance, specific risk factors for acquisition of ST131 are of practical utility; other studies have reported that older age, nursing home residency, urinary tract infections within 30 days, recent hospitalization (i.e. <3 months), and recent exposure to antimicrobial agents^29–32^ are independent predictors of infection with ST131. A profile of such risk factors, taking into account local microbiology and resistance profiles, can help inform appropriate and timely empirical treatment of infected patients.

To address the paucity of data in Southeast Asia and to discover risk factors for infection with ST131 *E*. *coli*, we undertook a multi-national, multi-centre study of *E*. *coli* bacteremia cases in Southeast Asia. As in other geographical areas, we found that ST131 *E*. *coli* was the most common sequence type of *E*. *coli* causing such infections, and we therefore focused on clinical risk factors and phenotypic resistance profiles associated with ST131 infections. We performed genome sequencing on all strains and examined the resistance genes, virulence factors, and phylogenetic relatedness of the ST131 strains. As expected, we found ST131 strains from all three major clades; interestingly, however, in Southeast Asia, C2/H30-Rx strains were all very closely related to each other; we refer to these strains as the SEA-C2 (Southeast Asia-C2) subclone of ST131. Remarkably, we found that the SEA-C2 subclone is solely responsible for the higher observed rates of invasive infection and ESBL-mediated antibiotic resistance associated with ST131 *E*. *coli* in Southeast Asia. We conclude that, for Southeast Asia, ST131 is dominated by a locally circulating clone, which may facilitate diagnosis and guide treatment of bacteremia patients in the region.

## Methods

### Study design and antimicrobial susceptibility testing

**Figure 1:**
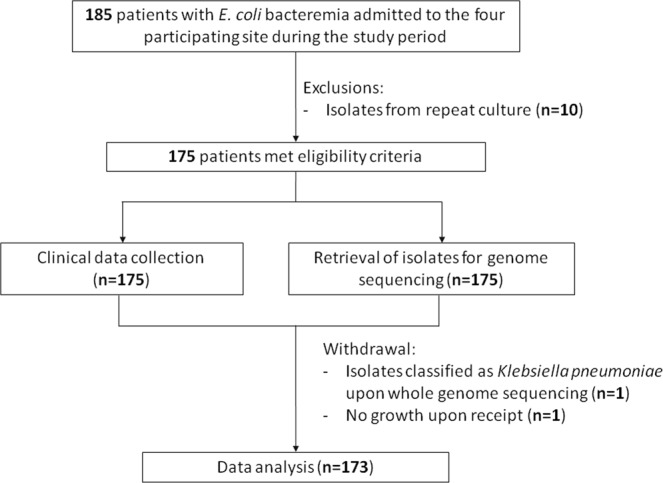
Flow chart of study samples. A flow chart for study enrollment and reasons for sample exclusion is shown.

Consecutive non-duplicate bacteremic isolates were collected from five hospitals in Southeast Asia (Tan Tock Seng Hospital (TTSH), National University Hospital (NUH), Singapore General Hospital (SGH), Singapore; Thammasat University Hospital (TUH), Thailand; and University Malaya Medical Center (UMMC), Malaysia) and sent to participating clinical microbiological laboratories for antimicrobial susceptibility testing. Strains were collected in July 2015 in the Singapore hospitals and from August to November 2015 in the hospitals in Thailand and Malaysia. Minimum inhibitory concentrations were determined with the VITEK system or E-test for the following antimicrobial agents: amikacin, gentamicin, ampicillin, amoxicilin-clavulanate, piperacillin-tazobactam, cefazolin, ceftriaxone, ceftazidime, cefepime, ciprofloxacin, trimethoprim-sulfamethoxazole, ertapenem, imipenem, and meropenem. The results were interpreted according to either the European Committee on Antimicrobial Susceptibility testing (EUCAST) (http://www.eucast.org) or the Clinical and Laboratory Standards Institute (CLSI) standards^33^, according to each hospital’s routine practice. Of note, efforts have been made to harmonize guidelines^34^, and specifically for *E*. *coli*, agreement between EUCAST and CLSI guidelines is poor only for amikacin among the antibiotics we tested^35^. Isolates that demonstrated complete or intermediate resistance to a given antimicrobial agent were considered non-susceptible. *E*. *coli* multidrug resistance (MDR) was defined as resistance to one or more agents in three or more classes of tested drugs^36^. 40 isolates were requested from each participating hospital. Some patients had multiple isolates; in these cases, the first isolate was chosen for inclusion. In total, 185 strains were collected, from which ten were excluded because they were not the first isolate from the patient; one was excluded because it was classified as *Klebsiella pneumoniae* upon whole genome sequencing; and one was excluded because the isolate did not grow upon receipt. The final set analyzed thus consisted of 173 strains (40 from TTSH, 39 from NUH, 40 from SGH, 18 from TUH, and 36 from UMMC). A flowchart of the samples is shown in Fig. 1.

### Data collection and definitions

Data collection included patient demographics (age and gender), underlying comorbidities (Charlson’s comorbidity score^37^), onset of infection (e.g., community onset, healthcare associated, nosocomial associated), antimicrobial susceptibility profile, source of bacteremia, severity of illness (APACHE II score^38^), antibiotic treatment (e.g., empiric and definitive antibiotics, duration of regimen), and outcomes (e.g., clinical and microbiological cure, mortality, recurrence). Empiric antibiotics were defined as those given to patients in the first 48 hours before antimicrobial susceptibility data were available, and definitive antibiotics were defined as those guided by the results of antimicrobial susceptibility testing. Clinical cure was defined as resolution of signs and symptoms of infection within seven days. Microbiological cure was defined as documented clearance of *E*. *coli* bacteremia within 30 days for a subset of patients with repeat blood cultures. Mortality was defined as all cause mortality at 30 days. Recurrence was defined as the presence of *E*. *coli* bacteremia after microbiological cure within 30 days.

The definition of healthcare-associated bacteremia was derived from a study in 2002^39^ with minor amendments. Community-acquired bacteremia was defined by a positive blood culture obtained at the time of hospital admission or within 48 hours after hospital admission for patients who did not fit the criteria for a healthcare-associated infection. Healthcare-associated infections were defined as: (i) a positive blood culture obtained from patients who had been hospitalized for 48 hours or longer; or (ii) a positive blood culture obtained at the time of hospital admission or within 48 hours of admission if the patient fulfilled any of the following criteria: hospitalized within 90 days before culture specimen collection; resident of a nursing home or long-term care facility (LTCF); received intravenous therapy at home within 30 days before the bacteremia; or received wound care, dialysis, and/or chemotherapy within 30 days before the bacteremia.

### DNA extraction and sequencing library preparation

Each isolate was streaked to single colonies on LB-agar. A single colony was inoculated into Luria-Bertani broth (Gibco) and cultured overnight at 37 °C with agitation. Cells from 1 ml of this culture were collected by centrifugation at 14,000 × g for 1 minute. Genomic DNA was isolated from the resulting pelleted bacteria using the QIAamp DNA mini kit (Qiagen). DNA samples were quantified using a QUBIT 2.0 fluorometer (Invitrogen). Sequencing libraries were prepared with the Nextera XT Library Prep Kit (Illumina) according to the manufacturer’s instructions. The adapters were indexed using either the Nextera XT Index Kit or the Nextera XT Index Kit v2 (Illumina). Finally, 10 nM of each sample DNA sequencing library were pooled together (giving a final concentration of 10 nM of the aggregate pooled library) and sequenced on a HiSeq 4000 (Illumina) with a 2 × 151 run.

Long read sequencing for ST131-TTSH-ECO-10 was performed as part of the Singapore PoreCamp (http://porecamp.github.io/singapore/). Genomic DNA for ST131-TTSH-ECO-10 was mixed in equal proportions (on a mass basis) with genomic DNA from two other bacterial strains with estimated GC content higher and lower than *E*. *coli* (which is approximately 50%). A sequencing library was prepared from this DNA mixture using a Rapid Sequencing Kit (SQK-RAD004) and sequenced on a FLO-MIN107 flow cell using a MinION Mk1 device, with MinKNOW v2.2. Basecalling was performed with Albacore v2.2.7.

Long read sequencing for ST131-TTSH-ECO-16 was performed using a Ligation Sequencing Kit 1D (SQK-LSK108) for library preparation and a FLO-MIN107 flow cell on a GridION running MinKNOW v2.2, with basecalling using Guppy v1.8.5.

### Genome sequence analysis

Raw FASTQ reads were used to call resistance genes, virulence factors, and (MLST) using SRST2 (version 0.2.0)^40^ with default settings. The ARGAnnot database^41^ supplied with SRST2 was used to identify resistance genes. For virulence factors, we used the VFDB database^42^, processed as recommended by the SRST2 documentation. The Achtman scheme was used to assign MLSTs^43^. Publicly available sequence data was downloaded from the Genbank Short Read Archive. A random sample of the Illumina data sets annotated as *E*. *coli* as of November 11, 2017 were downloaded and processed identically as described above (only half were used due to data size limitations). Metadata (strain name and country of isolation) were obtained from Genbank using the EDirect utilities (https://ftp.ncbi.nlm.nih.gov/entrez/entrezdirect/). Countries were classified into regions according to the United Nations Geographic Regions scheme (https://unstats.un.org/unsd/methodology/m49/). In total, 10,088 (of 17,262) *E*. *coli* short read data sets were downloaded. In addition, 2200 whole genome sequences from the Genbank RefSeq database (all sequences annotated as *E*. *coli* as of April 26, 2016) were downloaded. These were processed using the same databases mentioned above for resistance genes, virulence factors, and MLST, but the assemblies were processed using a custom BLASTN-based allele caller instead of SRST2. In total we had 1013 ST131 strains included in this analysis (36 from this study; 140 RefSeq assemblies; 837 public short read data sets).

To create phylogenetic trees, we used a reference-based analysis. The chromosome (excluding plasmids) of the *E*. *coli* ST131 strain EC958 genome^44^ was used as the reference. FASTQ files were mapped using bwa (version 0.7.10)^45^; indel realignment and SNP (single nucleotide polymorphism) calling was performed using Lofreq* (version 2.1.2) with default parameters^46^. An overall phylogenetic tree (for all 1013 strains) was made by calculating a dissimilarity matrix using SNPRelate^47^ and inferring a neighbor-joining tree using APE (version 3.5)^48^. Approximately maximum likelihood phylogenetic trees were inferred for smaller subsets of strains (<100); these were created using FastTree 2.1.8 with the –gtr and –nt command line options^49^. All phylogenetic trees were visualised with GGTREE 3.2^50^. All R packages were run in R (3.2.2) (https://www.R-project.org). Delineation of ST131 clades A, B, C1, and C2 were done based on matching strain identifiers with two previous reports^12,15^; there were no ambiguities in the topology (i.e. all clade A strains from the previous two papers were also phylogenetically closely placed in our trees, and not mixed in with other B, C1, or C2 strains, and similarly for strains from the other clades).

### Plasmid analysis

ST131-TTSH-ECO-10 was assembled with canu v1.3^51^ with the genomeSize = 15 m (due to its mixture with two other strains) and -nanopore-raw parameters. Assembled sequences belonging to ST131-TTSH-ECO-10 were identified by mapping the Illumina data to the final assembly, which resulted in only two assembled contigs of 5,160,494 and 181,654 nt. These two contigs were polished with pilon v1.22^52^ (with default parameters) using the Illumina data, and the 183 kb contig (after polishing) was then used for subsequent analysis.

ST131-TTSH-ECO-16 was assembled using a hybrid strategy with ONT and Illumina reads using Unicycler v0.4.7^53^. This resulted in two large assembled contigs of 5,268,201 and 232,458 nt and four additional small contigs less than 5 kb each. The four small contigs were ignored, and the 232 kb contig was used in subsequent analysis.

**Figure 2:**
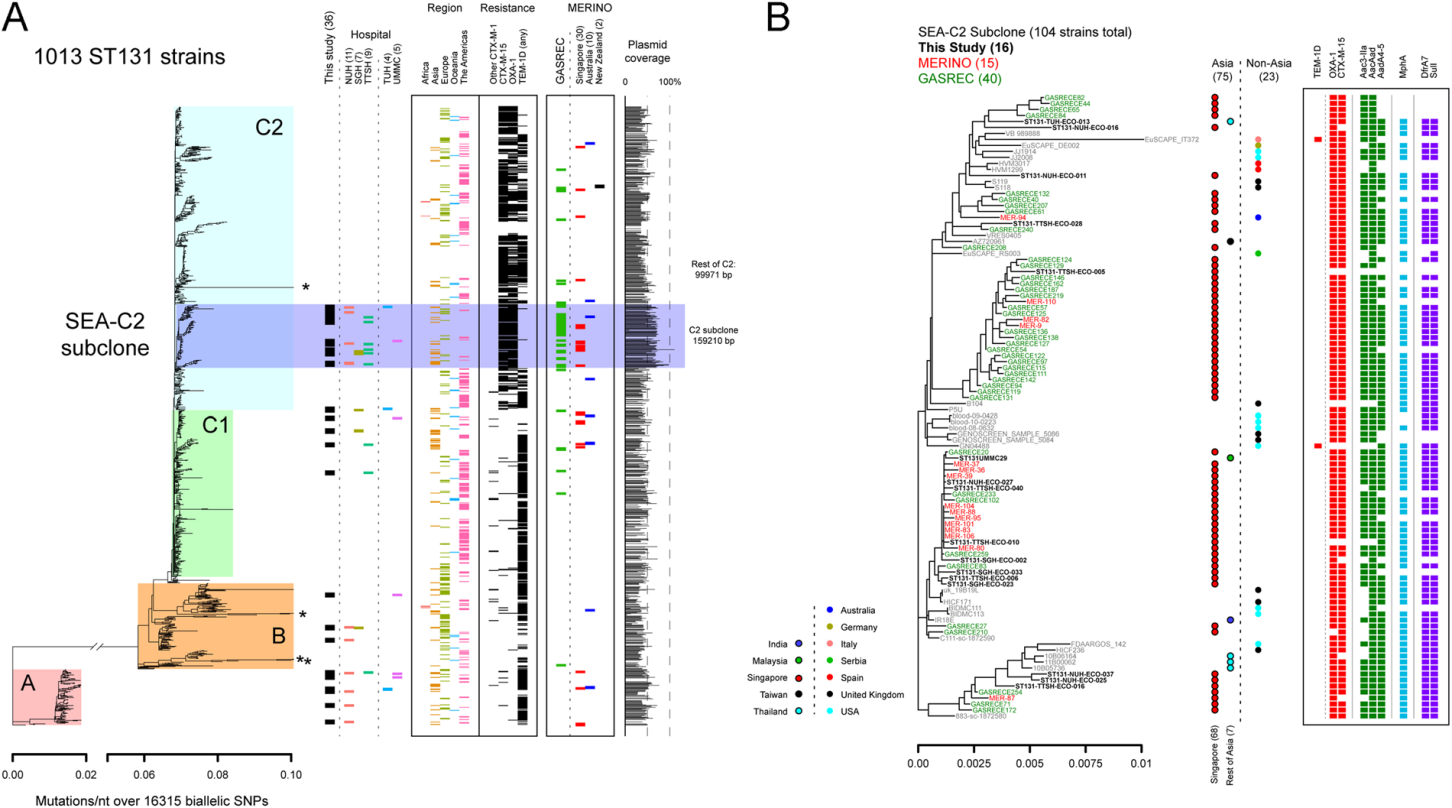
Phylogenomic analysis of ST131 strains (with removal of recombination). (**A**) Overview of all ST131 strains analyzed in this study. The tree on the left is an approximately maximum-likelihood tree, with mutations indicated on the x-axis at the bottom. Colored boxes indicate different subsets of the ST131 strains. From left to right, bars indicate the new strains contributed by this study (black boxes), the hospital from which the strains in this study were obtained (colored boxes below the “Hospital” label), the WHO region from which the strain was isolated (if available), resistance gene predictions for selected beta-lactamase genes, and the strains included from the GASREC and MERINO studies (with country of origin for MERINO strains). At the far right, the bar graph represents the percentage of the pTTSH16 plasmid that is covered by the assembly for each strain (based on blastn). Average plasmid coverage values for selected subsets of strains are indicated. (**B**) Expanded view of the SEA-C2 clone. Strains from this study, MERINO, or GASREC are indicated by font size and color; gray labels indicate other public data sets. Country of origin is indicated by the colored circles, with strains from Asia on the left with black outlines and strains from non-Asian areas on the right. Resistance gene predictions for each strain are indicated on the right with colored boxes; each class of resistance gene is in a separate color, with the gene indicated at the top.

The circular plasmid map was created using BRIG^54^. The default blast parameters for BRIG were used. The bar graph for Fig. 2A and the homology plots for Fig. S1 were generated using custom scripts. In brief, all strains were assembled using velvet v1.2.10^55^ with the VelvetOptimiser v2.2.4 helper script (https://github.com/tseemann/VelvetOptimiser). The resulting assemblies were analyzed with blastn v2.2.28+^56^ with default parameters, using the 232 kb pTTSH16 plasmid as a reference database. The bar graph in Fig. 2A represents the number of nucleotides in pTTSH16 that had any blast hit (all hits were >70% identity; >95% of the hits considered were >90% identity; and 89.3% of the hits considered were >99% identity). The plot of conservation in Fig. S1 represents the union of all blast hits reported for each strain to pTTSH16.

Conjugation-related genetic elements were predicted using oriTfinder^57^. The assemblies above were used in the web application. As a control, oriTfinder was used to predict conjugation-related genes in pEC958^58^, in which a single relaxase, a single Type IV coupling protein, and one Type IV secretion system locus were found.

Phage sequences were predicted using the PHASTER web tool^59^. Intact phage sequences were extracted from the summary and matched based on the given phage names and manual BLASTN analysis, which had perfect concordance.

### Statistical analysis

Comparisons between ST131 and non-ST131 isolates were evaluated using Chi square and Fisher exact tests. All tests were 2-sided. For comparisons of virulence factor prevalence, P-values were corrected by the Benjamini and Hochberg method^60^. P values <0.05 were considered statistically significant. The data were analyzed with STATA version 15 (Stata Statistical Software: Release 11, StataCorp LP, College Station, TX) and R (3.4.1).

### Ethics approval and consent to participate

The study was approved by the NHG Domain Specific Review Board (ref no: 2015/00777) in Singapore, UMMC Medical Ethics Committee (MED ID No 20158-1545) in Malaysia, and TUH Institutional Review Board in Thailand for the respective the study sites. All patients provided informed consent in accordance with the relevant regulations of the respective approval boards in each country. For patients under the age of 18, informed consent was obtained from a parent and/or legal guardian as per the relevant guidelines/regulations of the respective approval boards in each country. All research described was performed in accordance with relevant guidelines/regulations of the respective approval boards in each country.

## Results

### Collection of isolates

By collecting all bacteremia cases, regardless of resistance profiles, we were able to assess the association of MLST types with resistance profiles. A total of 5 hospitals in Southeast Asia participated by sending their first 40 consecutive *E*. *coli* bacteremia isolates. A total of 185 strains were sequenced using the Illumina platform; after excluding duplicate isolates from the same patient and eliminating mismatches in classification, we had a final data set of 173 sequenced *E*. *coli* bacteremia strains, with each strain representing a unique patient.

### Clinical features

Of the 173 patients, 77 (44.5%) were male, with a median age of 68 years (range 1–92). The median Charlson’s comorbidity score was 5 (0–13) and the median APACHE II score was 18 (2–58). Onset of bacteremia was community-associated in 81 patients (46.8%), leaving 92 that were healthcare-associated (53.2%). The top three sources of bacteremia were urinary (79 [45.7%]), primary (44 [25.4%]), and intra-abdominal (37 [21.4%]). Clinical cure was documented for 152 patients (87.9%), 30-day recurrence in 6 (3.47%), and 30-day mortality in 27 (15.6%). ST131 infections were similar to non-ST131 infections in terms of demographics, comorbidities, source of bacteremia, and clinical outcomes (Table 1).

**Table 1:** Patient clinical parameters, aggregated based on genomic analysis of infecting bacteria.

	Non-ST131 (137 strains, set A)		ST131 (36 strains, set B)		SEA-C2 clone (16 strains, set C)		ST131, not SEA-C2 (20 strains, set D)		All strains (173 strains)		P value (A vs B)	P-value (C vs D)	P-value (A vs D)
	Number	Percentage	Number	Percentage	Number	Percentage	Number	Percentage					
**Demographics**													
Age (years, median)	70	(1–96)	67.5	(0.25–91)	71	(53–91)	66.5	(1–96)	69	(0.25–96)	0.676	0.213	0.717
Gender (male)	64	46.72%	13	36.11%	6	37.50%	7	35.00%	77	44.51%	0.266	1.000	0.349
**Comorbidities**													
Charlson’s comorbidity score	5	(0–13)	6	(0–12)	5.5	(1–10)	6	(0–12)	5	(0–13)	0.187	0.979	0.339
Diabetes	67	48.91%	15	41.67%	8	50.00%	7	35.00%	82	47.40%	0.460	0.500	0.338
Renal disease	30	21.90%	8	22.22%	4	25.00%	4	20.00%	38	21.97%	1.000	1.000	1.000
Solid tumor	26	18.98%	9	25.00%	3	18.75%	6	30.00%	35	20.23%	0.485	0.700	0.247
Cerebral vascular disease	23	16.79%	5	13.89%	1	6.25%	4	20.00%	28	16.18%	0.803	0.355	0.752
Acute myocardial infarction	15	10.95%	5	13.89%	2	12.50%	3	15.00%	20	11.56%	0.571	1.000	0.705
**APACHE 2 (median**, **range)**	18	(2–58)	16	(6–34)	15	(11–33)	18	(6–34)	18	(2–58)	0.460	0.620	0.778
**Source of infection**													
Community	73	53.28%	8	22.22%	1	6.25%	7	35.00%	81	46.82%	**0**.001*	0.053	0.154
Healthcare associated	64	46.72%	28	77.78%	15	93.75%	13	65.00%	92	53.18%			
**Source of bacteraemia**													
Urinary	61	44.53%	18	50.00%	8	50.00%	10	50.00%	79	45.66%	0.578	1.000	0.811
Unknown (Primary)	35	25.55%	9	25.00%	4	25.00%	5	25.00%	44	25.43%	1.000	1.000	1.000
Intraabdominal	30	21.90%	7	19.44%	3	18.75%	4	20.00%	37	21.39%	0.823	1.000	1.000
Pulmonary	9	6.57%	3	8.33%	1	6.25%	2	10.00%	12	6.94%	0.716	1.000	0.633
Central venous catheter	2	1.46%	0	0.00%	0	0.00%	0	0.00%	2	1.16%	1.000	NA	1.000
Central nervous system	1	0.73%	0	0.00%	0	0.00%	0	0.00%	1	0.58%	1.000	NA	1.000
Others	6	4.38%	2	5.56%	1	6.25%	1	5.00%	8	4.62%	0.672	1.000	1.000
**Risk factors**													
Nursing home	4	2.92%	4	11.11%	3	18.75%	1	5.00%	8	4.62%	0.059	0.303	0.499
Recent hospitalisation within 90 days	49	35.77%	25	69.44%	13	81.25%	12	60.00%	74	42.77%	**0**.001*	0.277	**0**.049*
**Antibiotic exposure within 90 days**													
Carbapenems	6	4.38%	6	16.67%	5	31.25%	1	5.00%	12	6.94%	**0**.019*	0.069	1.000
Piperacillin-tazobactam	14	10.22%	6	16.67%	4	25.00%	2	10.00%	20	11.56%	0.377	0.374	1.000
Broad-spectrum cephalosporins	14	10.22%	11	10.22%	7	43.75%	4	20.00%	25	14.45%	**0**.006*	0.159	0.251
Fluoroquinolones	13	9.49%	10	27.78%	7	43.75%	3	15.00%	23	13.29%	**0**.010*	0.073	0.433
Cotrimoxazole	5	3.65%	1	2.78%	1	6.25%	0	0.00%	6	3.47%	1.000	0.444	1.000
Aminoglycosides	4	2.92%	0	0.00%	0	0.00%	0	0.00%	4	2.31%	0.581	NA	1.000
ICU admission prior to bacteraemia	7	5.11%	2	5.56%	2	12.50%	0	0.00%	9	5.20%	1.000	0.190	0.596
Dialysis within 30 days	3	2.19%	1	2.78%	0	0.00%	1	5.00%	4	2.31%	1.000	1.000	0.423
Surgery within 30 days	7	5.11%	0	0.00%	0	0.00%	0	0.00%	7	4.05%	0.347	NA	0.596
Central venous catheter within 30 days	13	9.49%	2	5.56%	0	0.00%	2	10.00%	15	8.67%	0.740	0.492	1.000
Urinary catheter within 30 days	23	16.79%	12	33.33%	4	25.00%	8	40.00%	35	20.23%	**0**.036*	0.481	**0**.030*
History of urinary infection	10	7.30%	11	30.56%	6	37.50%	5	25.00%	21	12.14%	**0**.001*	0.483	**0**.026*
Immunosuppression	48	35.04%	13	36.11%	5	31.25%	8	40.00%	61	35.26%	1.000	0.731	0.803
**Outcomes**													
Clinical cure	120	87.59%	32	88.89%	15	93.75%	17	85.00%	152	87.86%	1.000	0.613	0.723
Microbiological cure	107	78.10%	30	83.33%	15	93.75%	15	75.00%	137	79.19%	0.646	0.196	0.776
30-day recurrence	5	3.65%	1	2.78%	0	0.00%	1	5.00%	6	3.47%	1.000	1.000	0.565
30-day mortality	24	17.52%	3	8.33%	1	6.25%	2	10.00%	27	15.61%	0.208	0.686	0.532

*Indicates P < 0.05 (uncorrected); these are also set in bold font.

### Prevalence of ST131 in Southeast Asia

We used the sequencing data to infer MLST types. We found 56 distinct STs among the 173 isolates. Six of these STs accounted for 103 (59.5%) of 173 isolates. ST131 was the most common single ST, accounting for 36 (20.8%) of the isolates, followed by ST95 (23 isolates (13.3%)) and ST69 (16 isolates (9.2%)). The rest of the top 6 were ST38 with 10 (5.8%), ST1193 with 10 (5.8%), and ST73 with 8 (4.6%) isolates. We focused our subsequent analyses on the ST131 strains, as they represented the most common sequence type.

### One subclone of the C2 clade of ST131 accounts for nearly half of all ST131 in Southeast Asia

We analyzed the 36 ST131 strains in a global context using approximately half of the publicly available *E*. *coli* whole genome sequence data present in Genbank as of November 11, 2017. Metadata regarding the geographical location for each strain were available for 698/1013 strains; excluding the strains sequenced as part of this study (leaving 977 strains), isolates from Asia only represented 15.4% of all ST131 isolates (7.1% if the recent published GASREC (Genetic determinants of antimicrobial resistance and its impact on clinical response of bacteremia due to 3rd generation cephalosporin resistant *E*. *coli* and *K*. *pneumonia*)^61,62^ and MERINO (Meropenem versus piperacillin-tazobactam for definitive treatment of bloodstream infections due to ceftriaxone non-susceptible *Escherichia coli* and *Klebsiella spp*.)^21,63^ studies are excluded, see below), highlighting the generally lower sampling of strains from Asia.

Among the 36 new ST131 strains isolated in this study, all of the major ST131 clades (A, B, C1, and C2) were represented (Fig. 2A). As previously reported, the ESBL phenotype correlated with the presence of the CTX-M-15 gene in clade C2 strains, while CTX-M-9 genes were more common in non-C2 strains. While not an ESBL, the TEM-1D beta-lactamase is also prevalent among ST131 strains. Of further interest, we found that the OXA-1 ESBL was frequently also found in strains carrying CTX-M-15.

Interestingly, 16/36 of our ST131 strains all clustered relatively closely in a subclade of C2 strains. The branch leading to this subclade had a 94.2% bootstrap support, suggesting a monophyletic origin. Closer examination of this subclade (highlighted in dark blue in Fig. 2A; expanded view in Fig. 2B) suggested that many of these strains were isolated from Asia. Strains from this subclade mostly carried CTX-M-15 and OXA-1 ESBLs and were conspicuous for the low prevalence of TEM-1D. Interestingly, it has been noted that the introduction of CTX-M ESBLs into a geographic area tends to supplant existing TEM-class beta-lactamases^64^.

To verify that this subclade of C2 was truly overrepresented among ST131 strains from Asia, we examined the recently published GASREC data set of *E*. *coli* ceftriaxone-resistant bacteremia isolates, all from Singapore^61^. This data set included 124 total strains, of which 80 were *E*. *coli*; of these 80 *E*. *coli*, 57/80 (71.3%) were ST131 (including one single locus variant). We also examined the raw sequencing data for *E*. *coli* strains collected in the MERINO study, which included ESBL *E*. *coli* and *K*. *pneumoniae* strains from Singapore, Australia, and New Zealand^21^. Among the MERINO *E*. *coli* strains, 42/66 (63.6%) were ST131. Most of these 42 strains were from Singapore (30/42, 71.4%), with the rest from Australia (10) and New Zealand (2).

Overall, we found strong evidence for an overrepresentation of strains from Asia, and in particular from Singapore, within this subclade of C2 strains. This C2 subclade contained 75/186 (40.3%) of the strains from Asia, compared to 23/512 (4.5%) of the non-Asian strains (p < 2.2e-16, 2-tailed Fisher’s exact test). Even after removing all strains annotated as being from Singapore (including those from this study and in the publicly downloaded data sets), the C2 subclade contained 30 strains total, of which 7 were from Asia (23.3%); in contrast, the rest of the C2 clade contained 225 strains, of which 19 were from Asia (8.4%, p = 0.02033, 2-tailed Fisher’s exact test).

Among the collections with a strong Singaporean representation, the C2 subclade accounted for 16/36 (44.4%) of this study’s ST131 strains; 40/57 (70.1%) of the GASREC ST131 strains; and 14/42 (33.3%) of the MERINO ST131 strains. Only this study included strains from other countries in Southeast Asia, from which we found one strain each from Malaysia and Thailand (only one hospital from each of these countries was included). We therefore hereafter refer to strains within this C2 subclade as belonging to the “SEA-C2 clone”, which represents a previously undefined subset of C2 strains.

**Figure 3:**
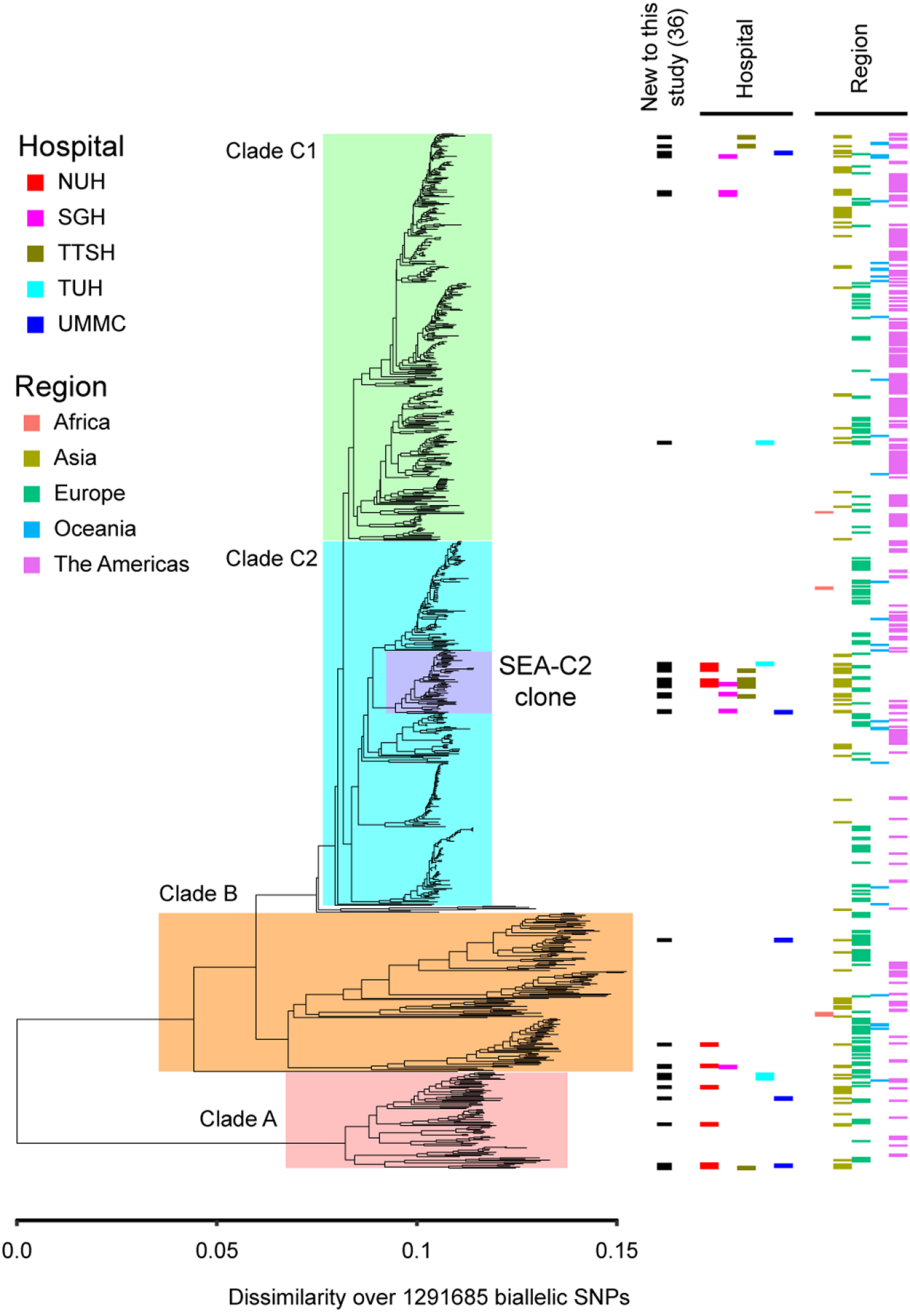
Phylogenomic analysis of ST131 strains (without removing recombination). A neighbor joining tree with all SNP positions is shown. Major clades of ST131 are highlighted by colored boxes. The SEA-C2 clone is highlighted in purple. From left to right, bars indicate the new strains contributed by this study (black boxes), the hospital from which the strains in this study were obtained (colored boxes below the “Hospital” label), and the WHO region from which the strain was isolated (if available). The SEA-C2 clone is still monophyletic in this analysis.

The above analysis, in which we found the SEA-C2 strains to be closely related and forming an apparently monophyletic subclone of the C2 clade, was performed after removing the recombination regions identified by Petty, *et al*.^12^; this reduced the phylogenetic analysis to 16,315 SNPs. Including all genomic regions, even when they may have undergone recombination, has the potential to alter the phylogenetic relationships as recombinant regions confound the “true” phylogenetic signal derived from vertically inherited sequence differences. It may also enhance the appearance of relatedness among strains if they share the same recombinant sequences. A neighbor-joining tree constructed from SNP distances (including all genomic regions) agreed with the previous result: the SEA-C2 clone strains were very closely related to each other, and they appeared to have a monophyletic origin (Fig. 3).

### The SEA-C2 clone is solely responsible for the higher beta-lactam resistance associated with ST131 in Southeast Asia

As expected, the ST131 strains isolated in this study were, overall, more antibiotic resistant than the non-ST131 strains. (Of note, a very low rate of carbapenem resistance (one non-ST131 strain) was found in this study).

ST131 isolates exhibited significantly higher prevalence of resistance (based on antibiograms) to beta-lactam, aminoglycoside, and fluoroquinolone antibiotics than non-ST131 strains (Table 2, compare sets A and B). Strains from the SEA-C2 clone were also highly resistant to these same antibiotics (Table 2, set C). Surprisingly, when SEA-C2 strains were excluded, the remaining ST131 strains were not significantly different in resistance to non-ST131 strains (Table 2, sets A and D).

**Table 2:** Antibiotic resistance phenotypes and resistance gene presence, aggregated based on genomic analysis of infecting bacteria.

Antibiotic	Non-ST131 (137 strains, set A)		ST131 (36 strains, set B)		SEA-C2 clone (16 strains, set C)		ST131, not SEA-C2 (20 strains, set D)		P value (A vs B)	P-value (C vs D)	P-value (A vs D)
	Number	Percentage	Number	Percentage	Number	Percentage	Number	Percentage			
Ampicillin	89	65.0%	29	80.56%	15	93.75%	14	70.00%	0.107	0.104	0.803
Amoxicilin-clavulanate	28	20.4%	16	44.40%	13	81.25%	3	15.00%	**0**.005*	**0**.000*	0.766
Piperacillin-tazobactam	9	6.6%	6	16.67%	5	31.25%	1	5.00%	0.089	0.069	1.000
Cefazolin/Cefalexin	48	35.0%	22	61.11%	13	81.25%	9	45.00%	**0**.007*	**0**.041*	0.457
Ceftriaxone	29	21.2%	21	58.33%	13	81.25%	8	40.00%	**0**.000*	**0**.019*	0.088
Ceftazidime	24	17.5%	18	50.00%	11	68.75%	7	35.00%	**0**.000*	0.078	0.077
Cefepime	22	16.1%	20	55.56%	12	75.00%	8	40.00%	**0**.000*	**0**.049*	**0**.028*
Imipenem	1	0.7%	0	0.00%	0	0.00%	0	0.00%	1.000	1.000	1.000
Meropenem	1	0.7%	0	0.00%	0	0.00%	0	0.00%	1.000	1.000	1.000
Ertapenem	0	0.00%	0	0.00%	0	0.00%	0	0.00%	**0**.000*	1.000	1.000
Gentamicin	17	12.4%	15	41.67%	11	68.75%	4	20.00%	**0**.000*	**0**.006*	0.313
Amikacin	4	2.9%	4	11.11%	4	25.00%	0	0.00%	0.059	**0**.031*	1.000
Ciprofloxacin	34	24.8%	25	69.44%	15	93.75%	10	50.00%	**0**.000*	**0**.009*	**0**.031*
Co-trimoxazole	61	44.5%	20	55.6%	12	75.00%	8	40.00%	0.264	**0**.049*	0.811
**Broad-spectrum cephalosporins**	30	21.9%	21	58.3%	13	81.25%	8	40.00%	**0**.000*	**0**.019*	0.095
**MDR resistance**	48	35.0%	24	66.7%	14	87.50%	10	50.00%	**0**.000*	**0**.032*	0.220
**Resistance Gene**											
CTX-M-15	4	2.92%	14	38.89%	12	75.00%	2	10.00%	**0**.000*	**0**.000*	0.169
Other CTX-M	14	10.22%	6	16.67%	0	0.00%	6	30.00%	0.377	**0**.024*	**0**.024*
OXA-1	8	5.84%	14	38.89%	14	87.50%	0	0.00%	**0**.000*	**0**.000*	0.393
OXA-48	1	0.73%	0	0.00%	0	0.00%	0	0.00%	1.000	1.000	1.000
TEM-1D	71	51.82%	11	30.56%	0	0.00%	11	55.00%	**0**.000*	**0**.001*	0.816
Other beta-lactamase^	9	6.57%	2	5.56%	1	6.25%	1	5.00%	1.000	1.000	1.000

* indicates P < 0.05 (uncorrected); these are also set in bold font.

^^^Includes AmpH, CMY, DHA.

These results for phenotypic antibiotic resistance were mirrored in our genomic analysis. Among the 173 *E*. *coli* isolates, 41 (24.7%) of *E*. *coli* isolates carried a gene encoding a CTX-M enzyme, most commonly CTX-M-15 (18/173, 10.4%) followed by CTX-M-27 (13/173, 7.5%). The gene encoding CTX-M-15 was significantly more prevalent among ST131 isolates (14/36, 38.9%) than non-ST131 isolates (4/137, 2.9%; p = 4.626e-08, 2-tailed Fisher’s exact test).

Numerous studies have associated CTX-M-15 with the C2 clade of ST131. Among the 36 ST131 strains in this study, CTX-M-15 was common and also mostly accounted for by C2 strains, all but one of which was in the SEA-C2 clone. We also found a higher prevalence of the OXA-1 gene among our ST131 strains. Interestingly, when we excluded the SEA-C2 strains, the remaining ST131 strains no longer had a significant enrichment of either CTX-M-15 or OXA-1 genes compared with non-ST131 strains (Table 2).

The TEM-1D beta-lactamase is neither an ESBL nor a carbapenemase, and therefore has not been a major focus of attention in previous ST131 studies. Intriguingly, we found that SEA-C2 strains generally do not carry the TEM-1D gene; the only exceptions are two strains that were isolated from the US and Europe. Similar patterns of antibiotic resistance gene presence/absence were observed in the GASREC (39/39 with CTX-M-15; 0/39 with TEM-1D) and MERINO (15/15 with CTX-M-15; 0/15 with TEM-1D) SEA-C2 strains.

### Strains in the SEA-C2 clone have a conserved plasmid

**Figure 4:**
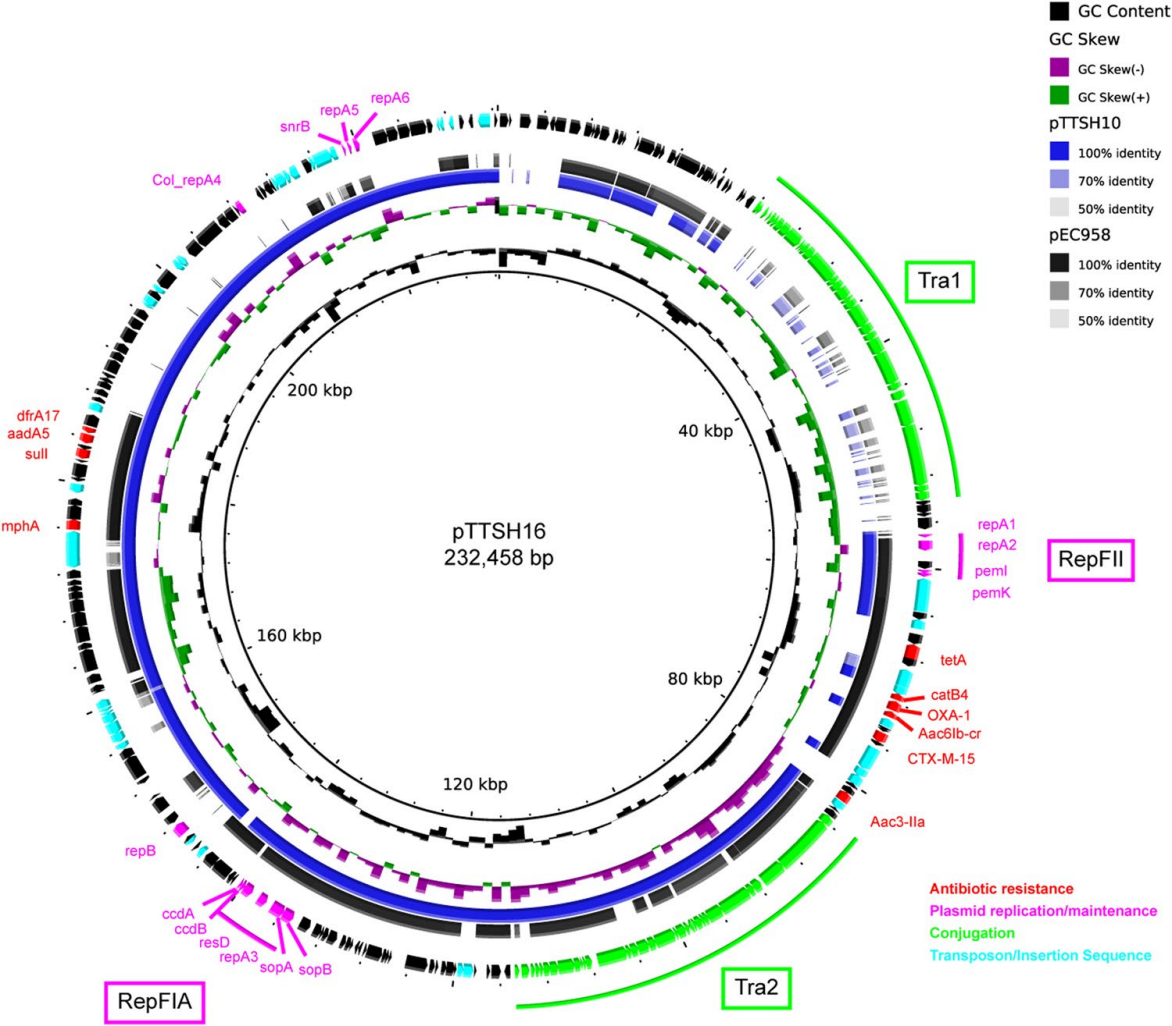
Similarity of SEA-C2 plasmids to the pEC958 ST131 plasmid. From inner to outer circle, the following are indicated: GC content, GC skew, pTTSH10, pEC958, gene annotation, and labels of selected genes/loci. The rings representing pTTSH10 and pEC958 consist of a colored bar indicating the sequence identity to pTTSH16 (used as the reference). Areas with less than 50% identity are considered not present and indicated by the absence of a colored bar in that section of the ring, as indicated by the legend at the top right. Genes with certain functional annotations are indicated in color, as indicated by the legend at the bottom right. Gene names for resistance genes and plasmid replication genes are indicated; loci containing conjugation-related genes and clusters of replication genes are annotated with a colored line and a boxed label.

We used long-read sequencing on two representative strains, ST131-TTSH-ECO-10 and ST131-TTSH-ECO-16, to definitively identify plasmid sequences. These strains differed in their beta-lactamase gene content; ST131-TTSH-ECO-10 lacked the CTX-M-15 and OXA-1 genes, while ST131-TTSH-ECO-16 had them (most of the SEA-C2 strains were similar to ST131-TTSH-ECO-16 in this respect) (Fig. 2B). We assembled 183,189 bp and 232,458 bp circular plasmids, respectively, designating them pTTSH10 and pTTSH16. Both plasmids appeared to have two copies of IncF replication sequences (denoted RepFIA and RepFII based on >97% blastn identity to the corresponding sequences in pEC958)^58^. They also carried four resistance genes in common: *aadA5* (aminoglycoside), *drfA17* (trimethoprim), *mphA* (macrolides), and *sulI* (sulfa). In addition, the pTTSH16 plasmid carried 6 additional resistance genes: *aac3-IIa* and *aac6Ib-cr* (aminoglycosides); *catB4* (chloramphenicol); CTX-M-15 and OXA-1 (ESBLs); and *tetA* (tetracycline). The online prediction tool oriTfinder (which predicts conjugation-related genes) identified a relaxase and Type IV conjugation protein in both pTTSH10 and pTTSH16. There was one type IV secretion system (T4SS) cluster in pTTSH10 but two T4SS clusters in pTTSH16, as seen in Fig. 4. Finally, an oriT transfer origin was only identified in pTTSH16.

Using the larger pTTSH16 plasmid as a reference, pTTSH10 shared 72.7% of the same sequence (using a cutoff of >90% nucleotide identity), with the major difference being the absence (in pTTSH10) of ~40 kb containing genes encoding a second set of conjugation-related proteins. Both of these plasmids are also similar to another well-characterized large pEC958 plasmid from the ST131 C2 strain EC958 (Fig. 4). The pEC958 plasmid showed the most difference in the two ~40 kb regions encoding conjugation machinery in pTTSH16.

Given the overlap in resistances identified in SEA-C2 strains with genes encoded on pTTSH16 (namely, CTX-M-15, OXA-1, *aadA5*, *aac3-IIa*, *aac6Ib-cr*, *dfrA17*, and *sulI*), we expected that most of the SEA-C2 strains would carry a similar plasmid. Using blastn to assess the coverage of pTTSH16 in assemblies of all the ST131 strains, we indeed found that the SEA-C2 strains had a significantly higher coverage of pTTSH16 (median 159,210 bp) compared with the rest of the C2 clade (99,971 bp) or all ST131 strains (98,912 bp) (Figs 2A and S1). We therefore conclude that the similar resistance profile among the SEA-C2 strains, particularly for ESBL genes, is driven by this conserved plasmid.

### The SEA-C2 clone is responsible for the higher virulence of ST131

ST131 strains have been reported to be proficient at causing UTI and bacteremia^9,65^. Our results are also consistent with generally higher virulence of ST131 strains. Similar to previous studies^29–32^, using a univariate analysis, we found the following clinical risk factors were associated with infection by an ST131 strain: healthcare-associated bacteremia; prior hospitalization within 90 days; urinary catheterization within 30 days; a history of urinary tract infections within 30 days before culture specimen collection; and recent exposure to carbapenems, piperacillin-tazobactam, or fluoroquinolones (Table 1). As seen with antibiotic resistances, when we removed strains from the SEA-C2 clone, we found no differences in any clinical parameters between patients infected by ST131 and non-ST131 strains.

### SEA-C2 strains have a similar virulence factor profile to other clade C2 strains

Given that SEA-C2 strains are driving the association between ST131 and virulence, we examined these strains for any associated virulence factor differences. We found several genes that are found together on a common ExPEC pathogenicity island, PAI-II^66–68^: *pap* genes encoding P pili; *hlyA* encoding a hemolysin; *cnf* encoding the cytotoxic necrotizing factor toxin; and the *tia*/*hek* outer membrane protein. These genes might be involved in the pathogenesis of the SEA-C2 clone; alternatively, the close relatedness of the SEA-C2 strains may mean this enrichment is due to a shared phylogenetic history. Indeed, overall, the pattern of virulence factor presence in the SEA-C2 clone was generally quite similar to that for other C2 ST131 strains (Fig. S2).

Looking within the SEA-C2 strains, we found that the *hlyABCD* operon, *cnf1*, and *tia/hek* genes were also unevenly distributed, and enabled differentiation of even further subdivisions of the SEA-C2 clone. One additional virulence factor, the *cdiA/B* contact-dependent inhibition system, was also significantly different among the subgroups of the SEA-C2 clone. These virulence factors were not uniformly conserved among the strains from Southeast Asia newly reported here. Therefore, while these genes are potential contributors to the expansion of the SEA-C2 strains in the region, the other possibility that their nonuniform distribution is the result of gene gain/loss events amplified by clonal expansion must be considered.

We also examined whether a unique phage profile might be associated with SEA-C2 strains. Using 4 complete or nearly complete SEA-C2 genomes (ST131-TTSH-ECO-10, ST131-TTSH-ECO-16, BIDMC111 (GCF_001030625.1), and BIDMC113 (GCF_001030675.1)) as well as a closely related non-SEA-C2 genome (BWH55 (GCF_001030365.1)), we found a largely conserved phage repertoire. Five intact phage loci were identified in common in all of these strains. Among the SEA-C2 strains, one additional phage was in common (the closest match was Enterobacteria phage mEp460, Genbank accession NC_019716, length 44.5 Kb). The SEA-C2 phage averaged 97.4% nucleotide identity over 29.6 Kb of the mEp460 sequence and contained intact genes encoding tail, protease, portal, terminase, lysin, and integrase proteins. The non-phage genes, which may have a role in virulence or fitness, encoded 6 hypothetical genes; methlyase and restriction nuclease proteins for the EcoRII restriction modification system; a DNA adenine methyltransferase; an arginyl-tRNA synthetase; a ClpP protease subunit; and a Lom outer membrane protein. All of these functions are also commonly found in other annotated Enterobacterial phages.

## Discussion

The recent global spread of the ST131 clone of *E*. *coli* is a remarkable example of the rapid expansion of a successful pathogen^3,8,9^. ST131 is made up of four predominant clades or sublineages: A, B, C1, and C2^8,12,15^. These can be differentiated by multiple genetic and some phenotypic methods, most accurately with whole genome phylogeny, specific gene allele differences (such as in *fimH*), and resistance gene profiles^9,15,69^. To date, the global success of ST131 has been largely due to the C1 and C2 clades, a pattern that has been verified by multiple investigators examining strains across multiple continents (see, for example^9,70^).

The bulk of data on ST131, however, remains confined to the Americas (dominated by the USA), Europe, and Oceania (mostly represented by Australia). Based on this broad international spread, it has been presumed that other geographical regions would show a similar pattern of ST131 prevalence^23^; this hypothesis has largely been validated as data has become available in Asia, Africa, the Middle East, and South America^9^. However, these other regions remain relatively undersampled, and the possibility remains for distinct patterns in the local microbiology. Indeed, hints of locally prevalent clones, possibly indicating local variation in selection pressures or transmission dynamics, have been reported for *E*. *coli* ST131^71^ as well as other bacteria (such as *Shigella sonnei*^72^, *Streptococcus pneumoniae*^73^, and *Salmonella* Typhi^74^).

In this work, the first international, prospective sampling of *E*. *coli* bloodstream isolates in Southeast Asia, we found that ST131, as in many other areas of the world, is the most prevalent sequence type of *E*. *coli*. We also found that the C2 clade of ST131 was responsible for most of the ST131 infections. Remarkably, in our study, all but one of the ST131 C2 strains were very closely related, forming a monophyletic subclone within C2 that we refer to as the SEA-C2 clone. As has been reported in other studies, the ST131 C2 strains were driving both higher virulence and higher antibiotic resistance rates among ST131 strains. Interestingly, removal of the SEA-C2 clone strains from our data set resulted in the other ST131 strains having no higher virulence or resistance than the non-ST131 *E*. *coli* strains captured in this survey.

The majority of our patients and strains were from Singapore, and therefore the large majority of SEA-C2 strains were also from Singapore. Furthermore, in two other surveys of *E*. *coli* isolates that have included Southeast Asia (the GASREC^61^ and MERINO^21^ trials), only Singaporean strains were included. In these two studies, the SEA-C2 clone again represented the great majority of the Singaporean C2 ST131 strains, but not of the Oceania strains from the MERINO study. In addition, by design, our study captured consecutive *E*. *coli* bloodstream isolates regardless of resistance pattern; in contrast, both the GASREC and MERINO trials included only bloodstream isolates that were resistant to 3^rd^-generation cephalosporins, and thus could not assess whether exclusion of the SEA-C2 clone strains also led to a similar rate of resistance between other ST131 strains and non-ST131 strains. Given previously published data on the overall resistance of H30-Rx/C2 strains in general^8,9^, however, we speculate that only within Singapore will the SEA-C2 clone be responsible for the higher resistance of ST131 strains overall. Finally, given the high representation of Singapore in this study, GASREC, and MERINO, the SEA-C2 clone is dominated by Singaporean isolates. Removing these from the data set, however, still leads to the result that strains isolated from Asia are overrepresented in the SEA-C2 clone.

While strains in the SEA-C2 clone were responsible for the association of ST131 with higher virulence and antibiotic resistance, the SEA-C2 clone itself seemed to be similar to other C2 strains overall in terms of these characteristics; indeed, virulence factor presence has been presumed to partially underlie the success of ST131, but no convincing examples have yet been described^9^. Clinically, ST131 infections in this study were associated with similar patterns of disease and risk factors to previous reports^29–32^: a history of urinary infections, healthcare-associated infections, and recent exposure to fluoroquinolones and third-generation cephalosporins. ST131 infections were three times more likely among healthcare-associated infections compared with community acquired infections; this contrasts with some early studies in which community acquired ST131 was more common^13,31,75–78^, but it agrees with other reports^29,79,80^. Healthcare-associated infection and a high Charlson’s score were independent risk factors for mortality in our study, similar to previous studies with mortality analysis^32,81^.

With respect to other genetic features of the SEA-C2 clone, we noted very similar antibiotic resistance and virulence factor profiles within the SEA-C2 clone, as would be expected for a closely related subclone of bacteria. The similarity in antibiotic resistance profiles was largely driven by a common plasmid found in SEA-C2 subclone strains, which is similar (with the exception of two conjugation-associated regions) to the well-described pEC958 multidrug resistance plasmid from the EC958 ST131 strain (itself a C2, but not SEA-C2, strain)^44,58^. Interestingly, nearly all strains in the SEA-C2 clone lacked a TEM-1D gene, which is common but not universally present in other C2 ST131 strains (of note, the TEM-1D gene is present on the pEC958 plasmid).

With respect to virulence factors, again there was high similarity among the SEA-C2 clone strains, which probably accounts for some of the genes being significantly overrepresented (supported by the close clustering of strains by genome-wide SNPs including potential recombination regions). Several of these genes are commonly found on a large pathogenicity island, called PAI-II, present in many ExPEC, particularly UPEC^67,68^. Of note, the *pap* gene locus is known to be important for strains to cause pyelonephritis, although the asymptomatic bacteriuria strain *E*. *coli* 83972 carries the *pap* operon and is used as a pre-emptive probiotic colonization strain in patients prone to urinary tract infection^82^. The *hlyA* hemolysin, in the context of urinary tract infection, is regulated by the Cpx stress response system and capable of inducing Caspase-1/Caspase-4 dependent inflammatory cell death *in vitro*; *in vivo*, overexpression of this toxin leads to rapid exfoliation of bladder epithelial cells, with the net effect of reducing bacterial burdens^83^. Expression of the Cnf1 toxin leads to a cytopathic effect in infected epithelial cells in cell culture, though there is conflicting data for its role during urinary tract infection in animal models^84,85^. Finally, the similar *tia* and *hek* adhesins are known to be important for invasion in intestinal pathogenic *E*. *coli*^86,87^ and NMEC^88^, respectively, but data for these has been limited to *in vitro* cell culture studies. Overall, while these virulence factors likely contribute to the virulence of strains that carry them, we suspect that their overrepresentation in the SEA-C2 clone (relative to the C2 strains in general) is probably due to the close phylogenetic relatedness of these strains. The fact that the SEA-C2 strains are similar to each other is consistent with a recent analysis of the accessory and regulatory genome regions of a diverse set of ST131 strains, which found multiple subtypes of C2 ST131 strains that share similar core and accessory genome features^89^. The SEA-C2 subclone differs, however, in that it appears to be more strongly geographically localized to SEA than the other C2 ST131 subtypes (which were noted to all be found in multiple continents and multiple host species)^89^.

## Conclusions

Our study is notable for examining strains in an undersampled geographical region (Southeast Asia) and regardless of resistance profile. This allowed us to characterize both the local microbiology in the region and the relative resistance rates among bacteremia strains. We have found that, like in other regions, the C2 clade of ST131 is contributing to the prevalence of ST131 infections and their higher antibiotic resistance rates, particularly for ESBL phenotypes. However, our study highlights a unique and previously undefined subclone of C2 strains, the SEA-C2 clone, which is highly prevalent in Singapore and significantly enriched in Asia in general. Many studies have provided strong evidence for an overall paradigm of ST131 being a globally disseminated clone^8,9,70,89^; however, we now show that Asia, and Singapore in particular, has a strong local bias in the C2 ST131 strains present. Given that Singapore is a highly connected transport hub, a prominent tourist destination, and a leading center for medical tourism, we speculate that there is ample opportunity for globally disseminated C2 ST131 strains to enter Singapore. (Of note, in Singapore, the NDM-1 carbapenemase is also being driven by local transmission of a single plasmid among multiple Enterobacteriaceae^90^, despite a similar opportunity for import of international NDM-1 carrying strains.) Therefore, further studies of the dynamics of strain importation and local transmission will be required to understand the reasons behind the prevalence of the SEA-C2 clone within Singapore and the region.

## Supplementary information

Supplementary data set

## Data Availability

All raw sequencing data described in this manuscript is available in the Genbank repository, under BioProject PJRNA488345.
