# Author Correction: The higher prevalence of extended spectrum beta-lactamases among *Escherichia coli* ST131 in Southeast Asia is driven by expansion of a single, locally prevalent subclone

**DOI:** 10.1038/s41598-021-87312-w

**Published:** 2021-04-14

**Authors:** Swaine L. Chen, Ying Ding, Anucha Apisarnthanarak, Shirin Kalimuddin, Sophia Archuleta, Sharifah Faridah Syed Omar, Partha Pratim De, Tse Hsien Koh, Kean Lee Chew, Nadia Atiya, Nuntra Suwantarat, Rukumani Devi Velayuthan, Joshua Guo Xian Wong, David C. Lye

**Affiliations:** 1 Genome Institute of Singapore, Agency for Science, Technology, and Research, 60 Biopolis Street, Genome #02-01, Singapore, 138672 Singapore; 2 Department of Medicine, Division of Infectious Diseases, Yong Loo Lin School of Medicine, National University of Singapore, 1E Kent Ridge Road, NUHS Tower Block, Level 10, Singapore, 119228 Singapore; 3 National Centre for Infectious Diseases, 16 Jalan Tan Tock Seng, Singapore, 308442 Singapore; 4 Division of Infectious Diseases, Faculty of Medicine, Thammasat University Hospital, 95 Phahonyothin Rd, Khlong Nueng, Khlong Luang District, Pathum Thani 12120 Thailand; 5 Department of Microbiology, Division of Pathology, Singapore General Hospital, Academia, Diagnostics Tower, Level 7, 20 College Road, Singapore, 169856 Singapore; 6 University Medicine Cluster, Division of Infectious Diseases, National University Hospital, 5 Lower Kent Ridge Road, Singapore, 119074 Singapore; 7 Department of Medical Microbiology, Faculty of Medicine, University of Malaya, 50603 Kuala Lumpur, Malaysia; 8 Communicable Diseases Centre, Institute of Infectious Disease and Epidemiology, Tan Tock Seng Hospital, Singapore, 308433 Singapore; 9 Duke-NUS Medical School, 8 College Road, Singapore, 169857 Singapore; 10 Chulabhorn International College of Medicine, Thammasat University, Pathum Thani, 12120 Thailand; 11 Lee Kong Chian School of Medicine, Nanyang Technological University, Singapore, 639798 Singapore; 12 Department of Infectious Diseases, Singapore General Hospital, Academia Level 3, 20 College Road, Singapore, 169856 Singapore; 13 Department of Laboratory Medicine, National University Hospital, 5 Lower Kent Ridge Road, Singapore, 119074 Singapore

Correction to: *Scientific Reports* 10.1038/s41598-019-49467-5, published online 13 September 2019

The original version of this Article contained a typographical error in the accession number in the Data Availability section where

“All raw sequencing data described in this manuscript is available in the Genbank repository, under BioProject PJRNA488345.”

now reads:

“All raw sequencing data described in this manuscript is available in the Genbank repository, under BioProject PRJNA488345.”

The original Article has been corrected.

