# Supplementary material for: The higher prevalence of extended spectrum beta-lactamases among Escherichia coli ST131 in Southeast Asia is driven by expansion of a single, locally prevalent subclone
Source: Sci Rep. 2019 Sep 13;9:13245. doi: 10.1038/s41598-019-49467-5 (PMC6744567; doi:10.1038/s41598-019-49467-5)
Supplement: Supplementary file 1 — Supplementary data set [file 41598_2019_49467_MOESM1_ESM.pdf]

Supplementary Information for:

The higher prevalence of extended spectrum beta-lactamases among Escherichia coli ST131 in Southeast Asia is driven by expansion of a single, locally prevalent subclone

Authors: Swaine L Chen, Ding Ying, Anucha Apisarnthanarak, Shirin Kalimuddin, Sophia Archuleta, Sharifah Faridah Syed Omar, Partha Pratim De, Tse Hsien Koh, Kean Lee Chew, Nadia Atiya, Nuntra Suwantarat, Rukumani Devi Velayuthan, Joshua Guo Xian Wong, David C Lye

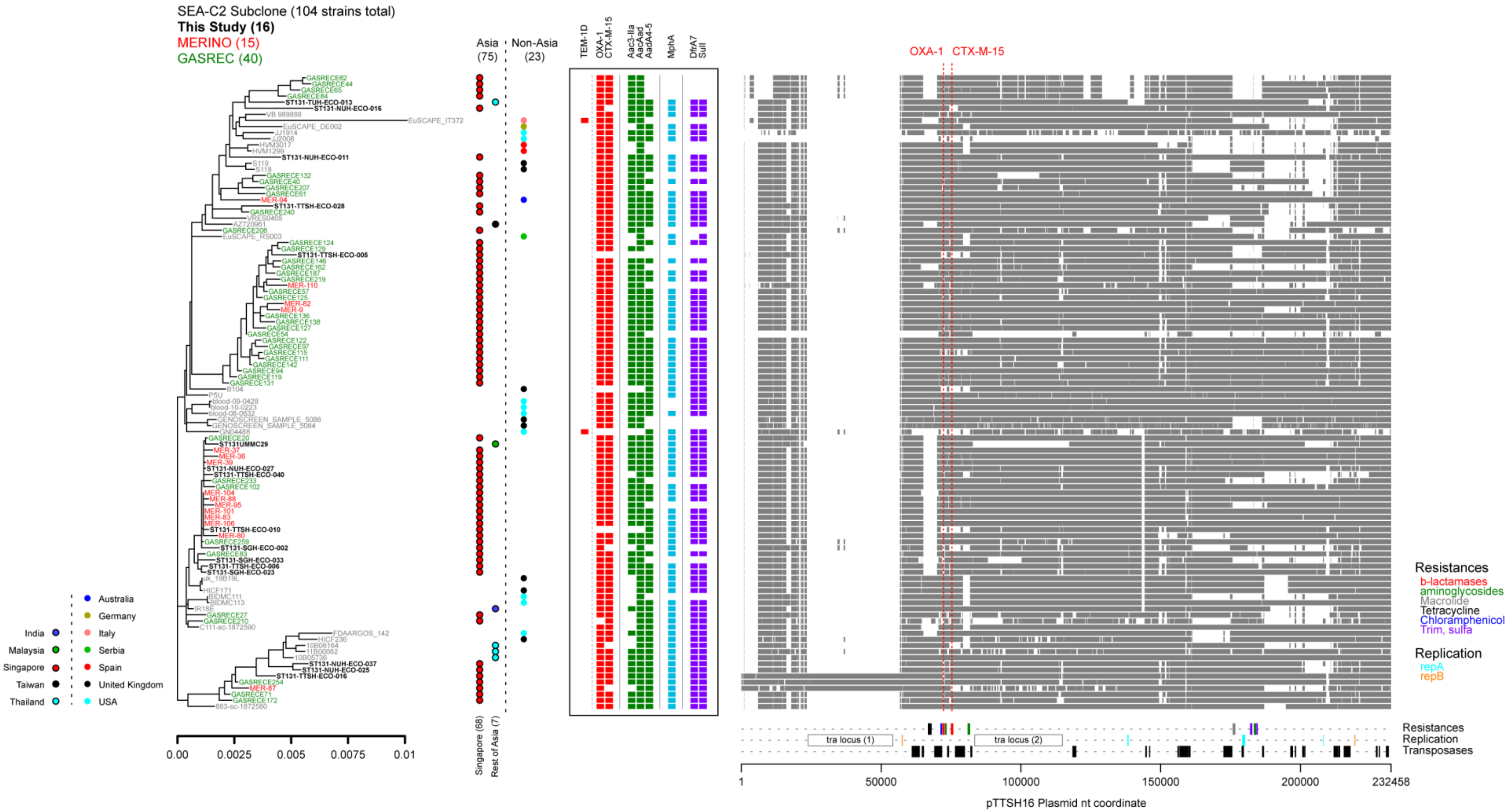

Figure S1. SEA-C2 strains share a conserved plasmid. The left side of the figure, including the phylogenetic tree, country of isolation, and resistance gene presence, is identical to that from Figure 2B and is shown for reference. On the right, areas of homology to pTTSH16 (based on blastn analysis of the assembled genome for each strain) are indicated by gray boxes. Nucleotide coordinates for pTTSH16 are represented on the x-axis. At the bottom, selected annotations are indicated for genes predicted to be involved in resistance, plasmid replication, conjugation, and mobile elements, with colors representing further functional predictions as indicated by the legend at the bottom right. Vertical red dotted lines indicate the locations of the OXA-1 and CTX-M-15 genes in the pTTSH16 sequence.

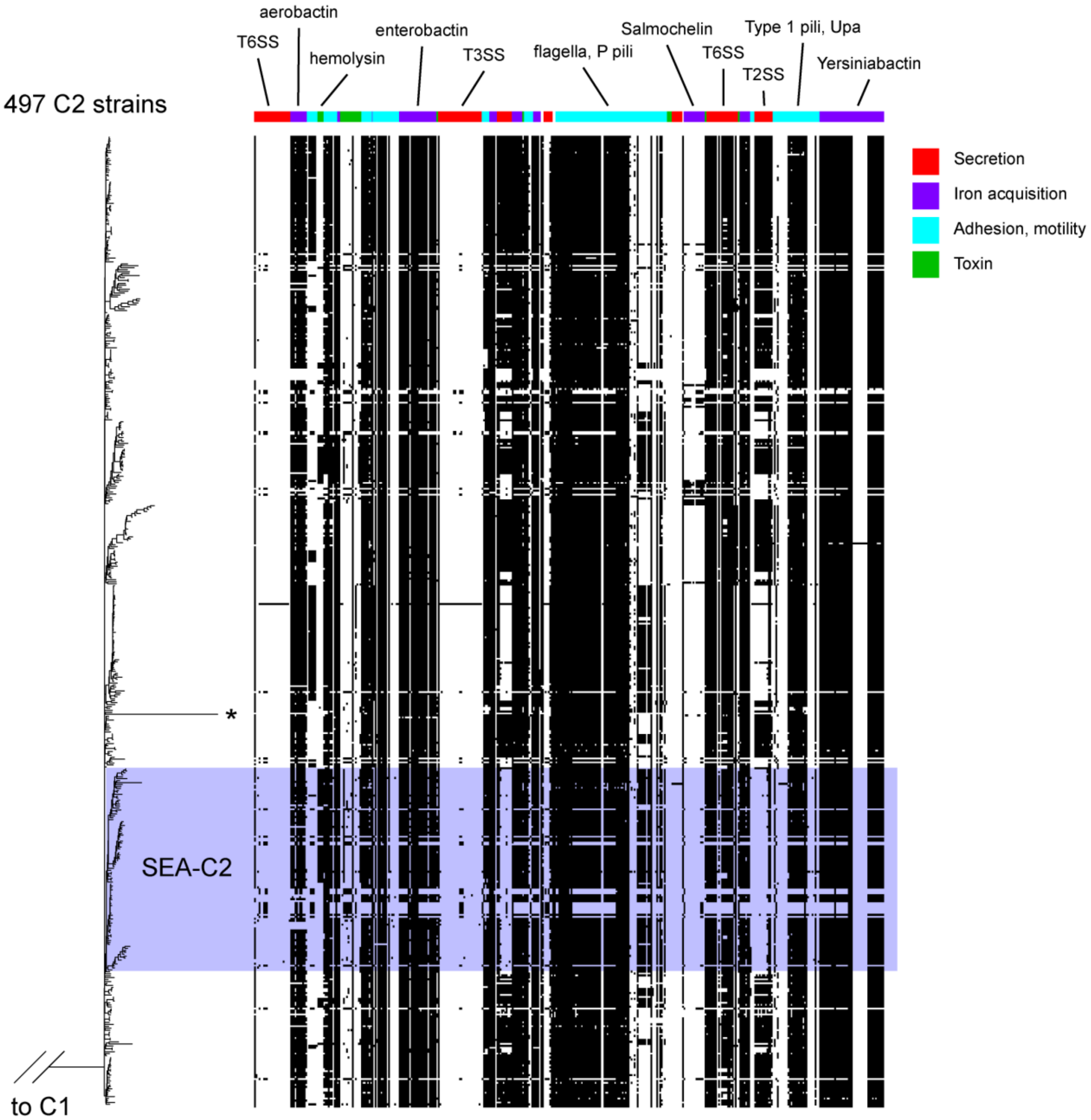

497 C2 strains
aerobactin
T6SS
hemolysin
enterobactin
T3SS
flagella, P pili
Salmochelin
T6SS
Type 1 pili, Upa
T2SS
Yersiniabactin
Secretion
Iron acquisition
Adhesion, motility
Toxin
*
SEA-C2
to C1

Figure S2. Virulence factor profiles for all ST131 C2 strains. The phylogenetic tree on the left is a subset of that shown in Figure 2A and is shown for reference. The SEA-C2 clone is highlighted in purple. The black boxes represent virulence factor presence, where each row corresponds to a strain and each column corresponds to one virulence factor. The colored bar at the top indicates a high level functional categorization of the virulence factors, with different colors representing different functional categories as indicated by the legend at the top right. Selected groups of virulence factors are also labeled at the top.
